# Glycine and GABA_A_ receptors mediate tonic and phasic inhibitory processes that contribute to prepulse inhibition in the goldfish startle network

**DOI:** 10.3389/fncir.2015.00012

**Published:** 2015-03-24

**Authors:** Paul C. P. Curtin, Thomas Preuss

**Affiliations:** ^1^Graduate Center, City University of New YorkNew York, NY, USA; ^2^Hunter College, City University of New YorkNew York, NY, USA

**Keywords:** sensorimotor integration, Mauthner cell, tonic inhibition, phasic inhibition, sensory processing, prepulse inhibition, auditory startle circuit

## Abstract

Prepulse inhibition (PPI) is understood as a sensorimotor gating process that attenuates sensory flow to the startle pathway during early stages (20–1000 ms) of information processing. Here, we applied *in vivo* electrophysiology and pharmacology to determine if PPI is mediated by glycine receptors (GlyRs) and/or GABA_A_ receptors (GABA_A_Rs) in the goldfish auditory startle circuit. Specifically, we used selective antagonists to dissect the contributions of target receptors on sound-evoked postsynaptic potentials (PSPs) recorded in the neurons that initiate startle, the Mauthner-cells (M-cell). We found that strychnine, a GlyR antagonist, disrupted a fast-activated (5 ms) and rapidly (<50 ms) decaying (feed-forward) inhibitory process that contributes to PPI at 20 ms prepulse/pulse inter-stimulus intervals (ISI). Additionally we observed increases of the evoked postsynaptic potential (PSP) peak amplitude (+87.43 ± 21.53%, *N* = 9) and duration (+204 ± 48.91%, *N* = 9). In contrast, treatment with bicuculline, a GABA_A_R antagonist, caused a general reduction in PPI across all tested interstimulus intervals (ISIs) (20–500 ms). Bicuculline also increased PSP peak amplitude (+133.8 ± 10.3%, *N* = 5) and PSP duration (+284.95 ± 65.64%, *N* = 5). Treatment with either antagonist also tonically increased post-synaptic excitability in the M-cells, reflected by an increase in the magnitude of antidromically-evoked action potentials (APs) by 15.07 ± 3.21%, *N* = 7 and 16.23 ± 7.08%, *N* = 5 for strychnine and bicuculline, respectively. These results suggest that GABA_A_Rs and GlyRs are functionally segregated to short- and longer-lasting sound-evoked (phasic) inhibitory processes that contribute to PPI, with the mediation of tonic inhibition by both receptor systems being critical for gain control within the M-cell startle circuit.

Startle is a rapid, massive contraction of facial and skeletal muscles that is triggered by the onset of intense and/or abrupt visual, auditory, or tactile stimuli. Startle is thought to function as a protective mechanism that minimizes impacts to vulnerable areas (e.g., the eyes) and/or facilitates collision avoidance or escape (Eaton et al., 1981; Bennett, 1984; Koch, 1999; Yeomans et al., 2002, 2006). The startle response is relatively stereotyped and predictably elicited, but is also the target of multiple modulatory mechanisms that enable dynamic adjustments to stimulus-response parameters in varying sensory and behavioral contexts. PPI (PPI) is a central inhibitory process that contributes to startle plasticity by briefly (20–1000 ms) reducing startle excitability while non-startling stimuli (prepulses) are processed (Graham, 1975; Hoffman and Ison, 1980; Koch, 1999). This sensorimotor gating mechanism is thought to preserve sensory processing and action selection by midbrain and forebrain processes that are activated by prepulses and would be disrupted by the subsequent initiation of startle (Graham, 1975). Consistent with this notion, information-processing disorders, including schizophrenia, Tourette’s syndrome, and obsessive-compulsive disorder are associated with diminished or disordered PPI (Swerdlow et al., 1992; Parwani et al., 2000; Braff et al., 2001). Consequently, identifying the neural processes underlying PPI presents an important goal for basic and translational neuroscience.

Anatomical and pharmacological studies indicate that PPI is mediated by multiple midbrain and forebrain circuits that modulate the time-course of inhibition in the startle circuit via multiple neurotransmitter systems. In mammals, startle is initiated by the firing of a population of giant hindbrain neurons in the ventrocaudal pontine reticular formation (PnC) (Lingenhöhl and Friauf, 1994; Fendt, 1999; Koch, 1999; Yeomans et al., 2002, 2006; Geis and Schmid, 2011). Anatomical studies indicate that PPI is produced by the excitation of midbrain circuits that project inhibitory terminals to PnC neurons; these include nuclei in the inferior colliculus, pedunculopontine tegmental nucleus, superior colliculus, and laterodorsal tegmental nucleus (Koch and Schnitzler, 1997; Fendt, 1999; Yeomans et al., 2010). Pharmacological studies in rodents emphasize that these inputs mediate PPI by multiple neurotransmitters that contribute discrete components toward the time-course of inhibition. Muscarinic receptors, for example, contribute to inhibition mediated at longer intervals, i.e., 100–1000 ms from prepulse onsets (Jones and Shannon, 2000; Ukai et al., 2004). GABA receptors are also critical mediators of PPI, with GABA_A_ receptors (GABA_A_Rs) contributing during the peak inhibitory response, and GABA_B_ receptors adding to the longer lasting inhibition mediated by muscarinic receptors (Yeomans et al., 2010). These lines of evidence derive from behavioral pharmacology studies in adult animals, or *ex vivo* slice preparations derived from immature tissue (e.g., Yeomans et al., 2010; Geis and Schmid, 2011).

The Mauthner-cell (M-cell) circuit in teleost fish presents an alternative model system for studying PPI and startle plasticity that is accessible to *in vivo* electrophysiology. The M-cells are a pair of large reticulospinal neurons, bilaterally opposed, that integrate excitatory and inhibitory inputs elicited by visual, auditory, and/or tactile stimulation (reviewed in Eaton et al., 2001; Korn and Faber, 2005). A single action-potential (AP) in either M-cell is sufficient to trigger a startle response (the C-start), and inhibition of APs is sufficient to prevent startle; thus, the M-cells are the decision-making sensorimotor interface for startle (Eaton et al., 1981). The M-cells are the focus of two well-characterized inhibitory networks that control startle excitability; these being, a collateral (feedback) inhibitory network that is bilaterally activated by cranial relay neurons when the M-cell fires, and a commissural (feed-forward) inhibitory network activated by parallel VIIIth nerve afferents to counter sound-evoked excitation in the M-cell and thereby regulate startle response properties (Eaton et al., 2001; Korn and Faber, 2005). Glycine receptor (GlyR) antagonists disrupt feedforward and feedback inhibition (Faber and Korn, 1978, 1987; Korn and Faber, 2005), but GABA_A_Rs also mediate M-cell excitability and are thought to be involved in auditory processing (Diamond et al., 1973). These inhibitory networks mediate two distinct types of processes: *phasic inhibition*, that includes transiently activated or stimulus-dependent inhibitory inputs, including the feed-forward and feedback inhibitory processes described, and *tonic inhibition*, that is, persistent inhibitory synaptic noise that arises from spontaneous quantal neurotransmitter release and intermittent firing at inhibitory synaptic terminals on the M-cell (Faber et al., 1989; Hatta et al., 2001; Marti et al., 2008).

A growing number of studies indicate that PPI in the M-cell system is modulated by multiple pre- and post-synaptic mechanisms. Neumeister et al. (2008) showed that PPI in goldfish is mediated by post-synaptic conductance changes activated in the M-cells. Burgess and Granato (2007) showed that dopaminergic agonists disrupt behavioral PPI in zebrafish, while Medan and Preuss (2011) showed dopaminergic mechanisms modulating time-specific components of PPI in the M-cell membrane, likely reflecting control of upstream networks involved in PPI. Furthermore, Curtin et al. (2013) showed that 5-HT_5A_ receptors modulate the excitability of goldfish M-cells, and linked these effects to changes in startle plasticity. Given these advances in our understanding of neuromodulatory processes contributing to startle plasticity, here we investigated the signaling mechanisms directly mediating PPI at the level of the M-cell.

This study focused on the roles of GlyRs and GABA_A_Rs in the mediation of PPI in the M-cells, the decision-making neurons of the goldfish auditory startle circuit. We targeted these receptor systems because they are densely expressed in the M-cell membrane (Triller et al., 1985; Seitanidou et al., 1988; Petrov et al., 1991; Lee et al., 1993; Sur et al., 1995) and their involvement in a diverse array of tonic and phasic inhibitory processes is well characterized (discussed above; see also Korn and Faber, 2005). We thus sought to identify the effector mechanisms for auditory PPI in the context of co-activated tonic and phasic inhibitory processes that are typically inaccessible in other model systems. Our findings indicate that GABA_A_Rs directly mediate the peak inhibitory components of PPI, while GlyRs indirectly contribute to the onset of PPI by the mediation of a feed-forward inhibitory process that overlaps with the earliest components of PPI.

## Materials and Methods

### Subjects

Sixteen common goldfish (*Carassius auratus*) of either sex were used in these experiments. Adult fish 7–13 cm in standard body length were purchased from Hunting Creek Fisheries (Thurmont, MD). Fish were socially housed, with 5–6 fish per 60L aquaria, in recirculating conditioned water (7.5 pH; 335 μS; 18°C) with a 12:12 light/dark photoperiod. Animals were housed and treated in accord with protocols established by the Institutional Animal Care and Use Committee (IACUC) of Hunter College, City University of New York.

### Electrophysiology

The surgical techniques and methods used for electrophysiological recordings were described previously (Medan and Preuss, 2011; Curtin et al., 2013). Fish were immersed in icewater for 10–15 min to induce immobility, then placed in a recording chamber. Two steel pins were placed on each side of the head to stabilize the fish and a tube was placed in the mouth to provide recirculating aerated water containing the general anesthetic, MS-222 (20 mg/l). This anesthetic dosage was chosen because prior studies have shown it does not interfere with auditory processing (Palmer and Mensinger, 2004; Cordova and Braun, 2007). Recirculating water in the recording chamber was initially near 0°C (when fish were fish removed from ice to begin procedures) but was gradually heated to 18°C before recordings were taken. Water conditions in all other measures were consistent with conditions in holding tanks.

A small lateral incision was made to expose the spinal cord at the caudal midbody, and a bipolar electrode was placed on the unopened spine to transmit low intensity (5–10 V) electrical stimulation generated by an isolated stimulator (Digitimer, Ltd, Wewyn Garden City, UK). A visible muscular contraction (twitch) was elicited with spinal stimulation to confirm proper placement of the spinal electrode, then d-tubocurarine (1 μg/g b.w.; Abbot, Chicago, IL) was administered intramuscularly. When the twitch response was abolished, typically within 0–3 min of injecting the tubocurarine, a craniotomy was performed to expose the medulla for microelectrode placement and recordings. The water level in the recording chamber was maintained throughout these procedures (and subsequent recordings) at the height of the mid-body, below the spinal incision.

The M-cell was localized by a characteristic negative extracellular potential (15–20 mV) generated in the axon cap during antidromic stimulation, which provides an unambiguous indicator of electrodes’ placement relative to the soma and axon cap (Faber and Korn, 1978). In these experiments, M-cells were impaled somatically with sharp electrodes (3–8 MΩ) filled with 5 M potassium acetate (KAc). An Axoprobe-1A amplifier (Molecular Devices, Foster City, CA) in current-clamp mode measured intracellular potentials, and a data acquisition card (PCI-E, National Instruments, Austin, TX) sampling at 25 KHz in a Macintosh G5 collected and recorded data. Recordings where resting membrane potential (RMP) varied by more than 10% from initial measure were excluded from analysis.

### Pharmacology

Our experimental design required the use of GlyR and GABA_A_R antagonists. We chose to use strychnine (Sigma-Aldritch), the classical GlyR antagonist, and bicuculline (Tocris Biosciences), the classical GABA_A_R antagonist, because both drugs have long and well-documented histories of use in the M-cell system. Drugs were dissolved in a 500 μL solution of physiological saline (in mM: 124.0 NaCl, 5.1 KCl, 2.8 NaH_2_PO_4,_ 0.9 MgSO_4_, 1.6 CaCl_2_, 5.6 glucose, and 20.0 HEPES, buffered to pH 7.2) and superfused directly to the exposed medulla, as in past studies (Diamond et al., 1973; Faber et al., 1991; Pereda et al., 1992, 1994; Hatta et al., 2001). This route of administration was chosen over others because it allowed direct reference to past studies and published dose-response curves. Given those studies, strychnine solutions (5 mM) were prepared as per Diamond et al. (1973), and bicuculline solutions (10 mM) were made as per Hatta et al. (2001). Physiological measures confirmed these concentrations were sufficient to achieve clear experimental effects (see Section Results). Nonetheless, given the 1.5 mm depth of the M-cell from the brain surface, and the diffusion volume, we expect that effective concentrations of strychnine and bicuculline were 1–2 orders of magnitude less at the site of the M-cell, following estimations offered in prior *in vivo* pharmacological studies with the M-cell system (Pereda et al., 1992, 1994; Hatta et al., 2001). All solutions were prepared on the day of experiments and were warmed to the temperature of the fish before application.

### Stimulus Protocols

Sound (pulse) stimuli were used to activate orthodromic inputs to the M-cells with or without a preceding sound stimulus (prepulse), the latter at multiple interstimulus intervals (ISIs) ranging between 20–500 ms. Sound stimuli in all experimental conditions were 200 Hz single-cycle “pips” produced at 80 dB re: 20 μPa in air. This stimulus intensity was chosen to elicit subthreshold responses in the M-cell because PPI is by definition elicited by subthreshold sounds, and the use of an identical conditioning (prepulse) and test (pulse) stimuli allows a within-subjects comparison of prepulse-pulse relationships on a trial-by-trial basis that is less sensitive to changes in baseline excitability. Stimuli were generated by a function generator (Agilent 33210A, Santa Clara, CA), and output to a shielded subwoofer (SA-WN250, Sony) placed 30 cm from the recording chamber. A microphone placed 10 cm above the fish’s head recorded acoustic waveforms and encoded these in parallel with intracellular recordings. A hydrophone (SQ01, Sensor, Collingwood, ON, Canada) was also used for sound calibration but was removed during experiments. In testing conditions that measured PPI, sound stimuli were produced at 6 inter-stimulus intervals (ISIs: 20, 50, 75, 150, 300, 500 ms) measured from the onset of each stimulus.

### Waveform Analysis of Evoked Synaptic Responses

Intracellular recordings were analyzed offline with custom and commercial software (Igor Pro; Wavemetrics, Lake Oswego, OR). To analyze the contribution of distinct inhibitory networks on sensory processing, we measured the peak depolarization and duration of sound-evoked post-synaptic potentials (PSPs) for comparison across treatment conditions. PSP duration was defined as the time between peak depolarization and the decline of excitation to 37% of peak, as per the calculation of tau (τ). We also compared peak depolarizations of early and later components of sound-evoked PSPs in order to compare how consistently and/or selectively each treatment condition acted on discrete temporal components of the sound response (see Section Results for details).

PPI was measured by comparing the peak depolarization evoked by an initial sound stimulus (prepulse) presented 20–500 ms prior to an identical stimulus (pulse), as in Lingenhöhl and Friauf (1994), Neumeister et al. (2008), Medan and Preuss (2011), Curtin et al. (2013). This method allows synaptic PPI effects to be quantified according to a commonly used formula 100 − (PSP_PULSE_/PSP_PREPULSE_* 100), i.e., as the normalized percentage change of the pulse response by a prepulse. Thus, higher PPI values reflect greater inhibition. Importantly, this relative measure allows the consistency of prepulse-pulse relationships to be tested across treatment conditions independently of possible changes in M-cell excitability (Medan and Preuss, 2011; Curtin et al., 2013). Both drugs increased spontaneous activity in the M-cell characterized by rhythmic bursts of sub-thresholdmembrane depolarizations and action potentials (APs), as previously described by Furukawa et al. (1964). For our analysis of the sound-evoked PSPs we excluded traces that involved such activity bouts. Spontaneous activity was identified in traces without sound stimuli as any deviation of membrane potential exceeding 10% of RMP. In traces with sound stimuli, spontaneous activity was identified as any depolarization initiated more than 10 ms from the onset of the sound stimuli.

### Statistical Analyses

Data were analyzed with JMP 10.0.0 (SAS) or Graphpad v5.0 (Prism). All data reported in figures and text reflect mean values and error bars illustrate SEM. All datasets were tested with the D’Agostino and Pearson Omnibus or K-S tests to confirm assumptions of normality were met for parametric statistical tests. In almost all cases, simple paired-*t* analyses were appropriate for the within-subjects design of these experiments. The exception to this was the analysis of the pharmacological effects on PPI, which by design considered three potential main effects (drug, ISI, and drug X ISI interaction), include two dimensions of repeated measures (ISI and drug treatment), and two axes that are better measured on continuous rather than categorical scales. Given those parameters, general linear mixed-models (GLMM) were used for those analyses. In these models subjects were treated as random effects (i.e., repeated measures) while ISI and drug-treatment were treated as fixed effects, and the dependent variable was the magnitude of PPI. Planned *post hoc* comparisons were made between comparable ISIs in drug and control conditions (e.g., %PPI at 20 ms ISIs before and after drug) with the Holmes-Bonferroni correction.

## Results

### Glycine Receptors Mediate Inhibition Contributing to the Onset of PPI

**Figure 1:**
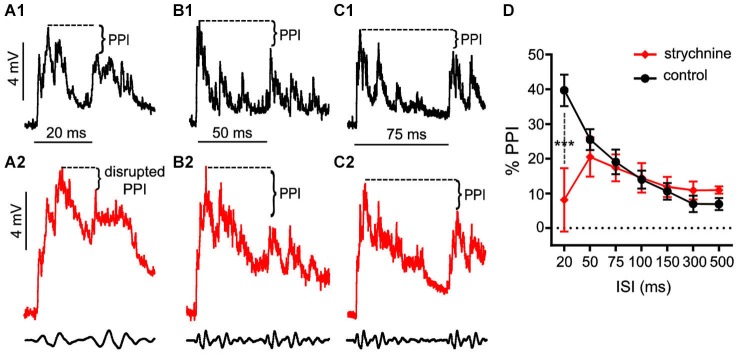
**Glycinergic inhibition contributes to the earliest components of PPI. (A–C)**. Sample intracellular recordings from the Mauthner-cell (M-cell) soma in response to paired (prepulse/pulse) sound pips at ISIs of 20 ms **(A1,A2)**, 50 ms **(B1,B2)**, and 75 ms **(C1,C2)** in control (black) and after application of the GlyR antagonist strychnine (red). Bottom traces show sound stimuli (200 Hz single-cycle “pips” at 80 dB re: 20 μPa). Dashed lines and brackets indicate how PPI was quantified by comparing peak depolarization between the two evoked post-synaptic potentials (PSPs). **(D)** Plots of the mean % PPI effect (± SEM, *N* = 9) across the full range of ISIs tested in control (black line) and strychnine (red line) conditions; asterisks indicate an ISI-specific reduction in PPI at the 20 ms ISI (see text).

These experiments tested if treatment with strychnine, a GlyR antagonist, affects auditory PPI in the Mauthner cells (M-cells), the decision-making neurons of the goldfish startle circuit. Figures 1A–C shows somatically recorded PSPs in response to prepulse/pulse sound stimuli (identical subthreshold 200 Hz “pips” at 80 dB; see methods) for ISIs ranging from 20–75 ms in control (black traces) and strychnine (red traces) treatment conditions. In each experiment (*N* = 9), 5–10 responses in a single M-cell were measured at ISIs ranging from 20–500 ms in control and drug treatment conditions. The results show an overall attenuation of the PSP to the secondary stimulus (pulse) when compared to the PSP evoked by the lead stimulus (prepulse) in control conditions; in short, synaptic PPI (Figures 1A–C). After application of strychnine synaptic PPI magnitude remained largely unchanged for all but the shortest ISI, despite the fact that the drug changed the overall PSP waveform (Figures 1A–C; black vs. red traces, see also below). Figure 1D plots the quantification of PPI across control (black line) and strychnine (red line) treatment conditions. Although PPI remained robustly intact after treatment with the GlyR antagonist for all ISIs >20 ms (Figures 1B,C, black vs. red traces; Figure 1D, black vs. red lines), we observed an ISI-specific reduction of PPI at the shortest ISI tested (20 ms; Figure 1A, black vs. red traces). Supporting these results, we found that strychnine had no significant main effect on the magnitude of PPI (*F*_(1,86.87)_ = 2.98, *P* = 0.088, *N* = 9), but our analysis identified a significant ISI X strychnine interaction (*F*_(6,83.68)_ = 5.7276, *P* < 0.0001, *N* = 9). *Post hoc* tests (Holmes-Bonferonni) confirmed this effect was due to an ISI-specific reduction in the PPI effect at the 20 ms ISI (*P* < 0.001). These findings indicate that GlyRs mediate inhibition that contributes to PPI for as long as 20 ms, but this glycinergic component decays within 50 ms of the onset of prepulse stimuli.

### Glycine Receptors Shape Auditory Processing via Multiple Mechanisms

**Figure 2:**
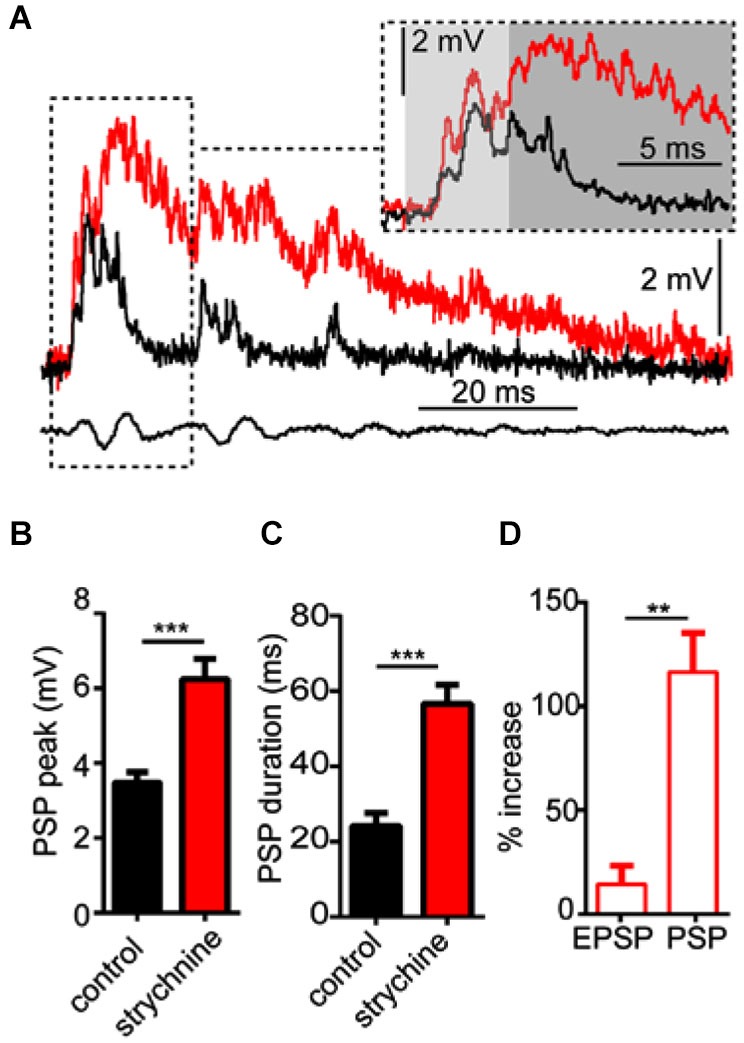
**Glycinergic inhibition mediates auditory processing in the M-cell. (A)** Sample intracellular recordings from the Mauthner-cell (M-cell) soma in response to an individual sound pip before (black trace) and after (red trace) treatment with strychnine. The insetshows the initial part of the evoked response at an expanded time scale. Light and dark shaded areas distinguish the initial (EPSP; 0–5 ms) and later components (mixed PSP; >5 ms) of the response, respectively. Bottom traces show sound stimulus (200 Hz single-cycle “pip” at 80 dB re: 20 μPa). **(B)** Plots of mean (± SEM, *N* = 9) overall peak amplitude of sound-evoked depolarization in control (black bar) and strychnine (red bar) conditions. **(C)** Plots of mean (± SEM, *N* = 9) PSP duration (Tau) before (black) and after (red) treatment with strychnine. **(D)** Plots of the mean (± SEM, *N* = 9) relative change in sound-evoked depolarization for the initial (EPSP) and later parts (mixed PSP) of the sound response after treatment with strychnine. Asterisks indicate *p* values in statistical comparisons that were < 0.01 (**), < 0.001 (***).

As noted above, strychnine also affected the waveform of sound evoked M-cell PSPs, i.e., auditory processing. In order to analyze this more directly, we tested the effects of strychnine on M-cell sound-evoked PSPs evoked by a single sound pip (i.e., independent of PPI). Figure 2A shows sample sound responses recorded in control (black trace) and strychnine (red trace) treatment conditions. The overall peak of PSPs increased on average by 87.43 ± 21.53% (*N* = 9; see Figure 2B) after treatment with strychnine. We confirmed this increase was statistically significant by paired-*t* test (*t*_(8)_ = 6.08, *P* = 0.0003, *N* = 9). The duration of sound-evoked PSPs (defined as per the calculation of τ; see Section Materials and Methods) was also significantly greater (204.0 ± 48.91% increase; *t*_(8)_ = 6.11, *P* = 0.0003, *N* = 9; Figure 2C) after treatment with strychnine.

We next analyzed how the GlyR antagonist acted on different components of the overall sound response. The M-cell PSP reflects the integration of multiple excitatory and inhibitory inputs activated by primary auditory afferences. These include electrotonic and chemical excitation via mixed VIIIth nerve synapses at the M-cell lateral dendrite (Furshpan, 1964; Faber and Korn, 1975; Lin and Faber, 1988a; Curti and Pereda, 2004). This excitation is counteracted by chemical inhibition (onset of about 5 ms relative to stimulus onset; see Preuss and Faber, 2003; Medan and Preuss, 2011) that peaks at 10–12 ms, mediated by a feed-forward network that is also activated by VIIIth nerve afferents (Korn and Faber, 2005; Szabo et al., 2006; Weiss et al., 2008; see also Introduction). In other words, the monosynaptic excitatory pathway and disynaptic inhibitory pathway allow a brief interval within the first 5 ms of the postsynaptic response when sound-evoked depolarization reflects largely excitatory inputs (i.e., an EPSP), whereas later components of the sound-response represent the integration of excitatory and inhibitory inputs (i.e., a mixed PSP). Figure 2A (inset) shows the onset of the sound-response in an expanded time scale to emphasize the effects of strychnine (compare black vs. red traces) on the EPSP (light gray area, 0–5 ms from stimulus onset) and the PSP (dark gray area, >5 ms from stimulus onset). We found that strychnine caused a relatively mild enhancement (14.33 ± 7.2% increase, *N* = 9) of peak depolarization during the initial EPSP, but a significantly greater enhancement of excitation during the mixed-PSP component of the response(116.5 ± 18.73% increase; *t*_(8)_ = 8.54, *P* = 0.0034; see Figure 2D). This time course suggests a minor effect of strychnine on presynaptic excitatory pathways and/or M-cell tonic excitability, but is consistent with a drug-induced disruption of feed-forward inhibition in the M-cell.

### GABA_A_ Receptors Mediate Peak Inhibitory Components of PPI

**Figure 3:**
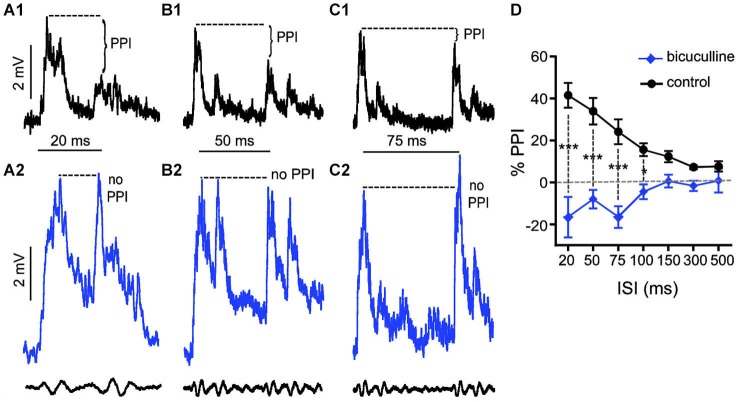
**GABA_A_Rs mediate peak inhibitory components of PPI. (A–C)** Sample intracellular recordings from the Mauthner-cell (M-cell) soma in response to paired (prepulse/pulse) sound pips at ISIs of 20 ms **(A1,A2)**, 50 ms **(B1,B2)**, and 75 ms **(C1,C2)** in control (black) and after application of the GABA_A_R antagonist bicuculline (blue). Bottom traces show sound stimuli (200 Hz single-cycle “pips” at 80 dB re: 20 μPa). Dashed lines and brackets indicate how PPI was quantified by comparing peak depolarization between the two evoked post-synaptic potentials (PSPs). **(D)** Plots of the mean % PPI effect (± SEM, *N* = 5) across the full range of ISIs tested in control (black line) and bicuculline (blue line) conditions; asterisks indicate an ISI-specific reduction in PPI at ISIs of 20, 50, 75, and 100 ms (see text).

We applied the same experimental approach as described above to test the effects of bicuculline (10 mM superfusion), a GABA_A_R antagonist, on auditory PPI at the level of the M-cells. Figures 3A–C shows sample intracellular M-cell recordings at three ISIs before (black traces) and after (blue traces) bicuculline treatment. As in experiments with strychnine, in these experiments (*N* = 5) sound-evoked excitation was measured in single M-cells for 5–10 trials at varying ISIs before and after treatment with an antagonist. Similar to the effects we observed with strychnine, we found that the GABA_A_R antagonist caused prominent changes in the amplitude and duration of sound evoked M-cell PSPs; these effects are analyzed in detail below. In contrast to the effects of strychnine, however, we found that treatment with bicuculline severely disrupted PPI. This was apparent in the overall reduction in PPI over the entire range of ISIs tested (see Figure 3D), and in ISI-specific effects where the reduction of PPI was most pronounced. Figures 3A–C shows traces at 20, 50, and 75 ms ISIs where the disruption of PPI was most prominent, i.e., both prepulse and pulse stimuli evoked similar levels of depolarization in drug conditions. In Figure 3D, the quantification of PPI is plotted across the range of ISIs tested. Our analysis of these data (GLMM) identified significant main effects of bicuculline treatment (*F*_(1,50.96)_ = 89.3722, *P* < 0.0001, *N* = 5) and the interaction of bicuculline x ISI (*F*_(6,50.59)_ = 7.4119, *P* < 0.0001, *N* = 5). *Post hoc* analyses (Bonferonni-Holm corrected) found the latter effect was attributable to ISI-specific reductions in PPI intensity at ISIs of 20 ms (*P* < 0.001), 50 ms (*P* < 0.001), 75 ms (*P* < 0.001), and 100 ms (*P* < 0.05). In sum, these results indicate that blockade of GABA_A_Rs causes a general disruption of PPI.

### GABA_A_ Receptors Mediate a Tonic Increase in Sound-Evoked Excitation

**Figure 4:**
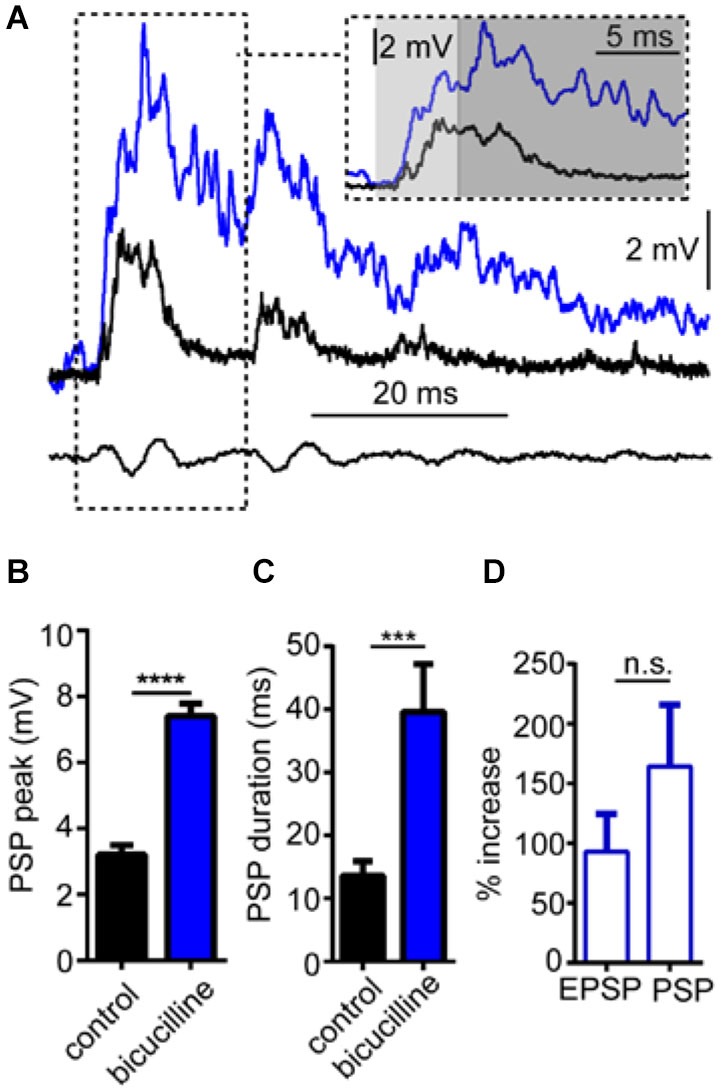
**GABA_A_Rs mediate auditory processing. (A)** Sample intracellular recordings from the Mauthner-cell (M-cell) soma in response to an individual sound pip before (black trace) and after (blue trace) treatment with the GABA_A_R antagonist bicuculline. The inset shows the initial part of the evoked response at an expanded time scale. Light and dark shaded areas distinguish the initial (EPSP; 0–5 ms) and later components (mixed PSP; >5 ms) of the response, respectively. Bottom traces show sound stimulus (200 Hz single-cycle “pip” at 80 dB re: 20 μPa). **(B)** Plots of mean (± SEM, *N* = 5) overall peak amplitude of sound-evoked depolarization in control (black bar) and bicuculline (blue bar) conditions. **(C)** Plots of mean (± SEM, *N* = 5) PSP duration (Tau) before (black) and after (blue) treatment with bicuculline. **(D)** Plots of the mean (± SEM, *N* = 5) relative change in sound-evoked depolarization for the initial (EPSP) and later parts (mixed PSP) of the sound response after treatment with strychnine. Asterisks indicate *p* values in statistical comparisons that were < 0.001 (***), < 0.0001 (****).

In these experiments, we tested the effects of the GABA_A_R antagonist, bicuculline on sound-evoked depolarization in separate trials using only a single sound pip (without prepulses) to determine how GABA_A_Rs contribute to auditory processing independently of PPI. We again approached our analysis by examining the effect of the GABA_A_R antagonist on the overall peak depolarization and duration of sound-evoked excitation. Figure 4A shows sound-evoked PSPs recorded in control (black trace) and bicuculline (blue trace) treatment conditions. We found that bicuculline significantly increased the mean overall peak of sound-evoked PSPs by 133.8 ± 10.3% (Figure 4B; *t*_(4)_ = 28.12, *P* < 0.0001, *N* = 5). Similarly, the duration of sound-evoked PSPs increased by 284.95 ± 65.64% in drug conditions (Figure 4C; *t*_(4)_ = 3.07, *P* = 0.037, *N* = 5).

As in our analysis of GlyR-mediated components of sound-evoked excitation, we also measured the potentially differential effects of the GABA_A_R antagonist on initial (EPSP) and subsequent components (PSP) of the sound-response (Figure 4A inset, light gray shading vs. dark gray shading). In contrast to strychnine, bicuculline produced large enhancement in both components of the response (EPSP: 90.95% increase; PSP: 164.32% increase); however, bicuculline’s effect on the EPSP was not significantly different from the PSP (*t*_(4)_ = 1.116, *P* = 0.327, *N* = 5). The latter result is consistent with a drug induced increase in presynaptic excitation and/or by a decrease in inhibitory tone in the M-cell system which increases the neurons excitability (see below).

### Strychnine and Bicuculline Disrupt Tonic Inhibition Contributing to M-cell Excitability

**Figure 5:**
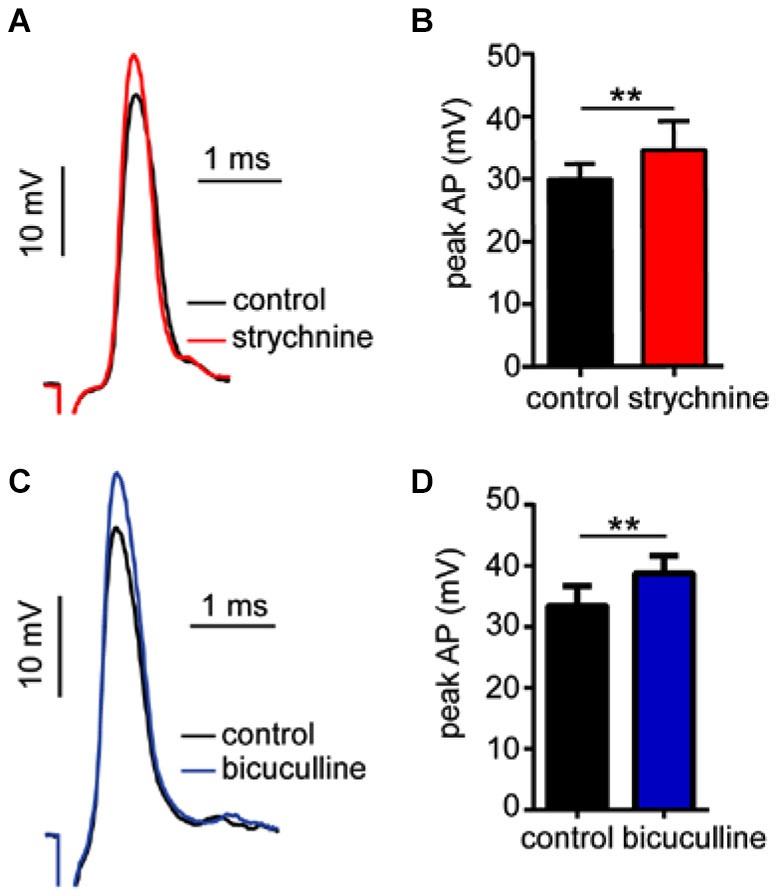
**Strychnine and bicuculline increase Mauthner-cell excitability. (A)** Sample recordings showing antidromically-evoked Mauthner-cell (M-cell) action-potentials (APs) in control conditions(black trace) and after treatment with strychnine (red trace, superimposed). **(B)** Plots of mean (± SEM) AP magnitude in control (black bar) and strychnine (red bars) treatment conditions. **(C)** Sample recordings of antidromically-evoked M-cell APs, in control (black trace) and bicuculline (blue trace, superimposed) treatment conditions. **(D)** Plots of mean (± SEM) AP magnitude in control (black bar) and bicucilline (blue bar) conditions. Asterisks indicate *p* values in statistical comparisons that were < 0.01 (**).

In prior experiments, we reported that GlyR and GABA_A_R antagonists enhance sound-evoked excitation in the M-cell (Figures 2, 4). Namely, strychnine treatment predominately enhanced later parts of the PSP, i.e., demonstrating a time-dependent effect, whereas bicuculline produced an enhancement of the entire PSP consistent with a tonic change in M-cell excitability. Accordingly, we next tested whether these antagonists affect electrotonic excitability in M-cell. Since the M-cells’ soma-dendritic membrane is non-regenerative, changes in the magnitude of somatic APs provide a measure of corresponding changes in tonic membrane conductivity (excitability; reviewed in Korn and Faber, 2005; Curtin et al., 2013). Figure 5A shows sample traces of APs elicited in control (black trace) and strychnine (red trace) treatment conditions. On average, treatment with strychnine increased the peak magnitude of APs by 15.07 ± 3.21% (Figure 5B; paired-*t*, *t*_(6)_ = 4.314, *P* = 0.005) consistent with a decrease in conductance. Importantly, RMP (RMP_control_ = −80.6 ± 0.82 mV; RMP_strychnine_ = −80.9 ± 0.86 mV) was not affected by treatment with strychnine (*t*_(9)_ = 0.4104, *P* = 0.69), indicating the disruption of a shunting inhibition rather than a persistent hyperpolarization.

Similarly, bicuculline treatment increased the magnitude of APs by 16.23 ± 7.08% (Figure 5C, black vs. blue traces; Figure 5D, *t*_(4)_ = 3.09, *P* = 0.036, *N* = 5), but had no significant effect on RMP (RMP_control_ = −78.6 ± 1.55 mV; RMP_bicuculline_ = −77.22 ± 1.35 mV), consistent with a shunting inhibitory process.

In sum, these results are consistent with the notion that tonic inhibitory processes regulate M-cell excitability, and these processes are mediated by GlyRs and GABA_A_Rs.

## Discussion

The aim of this study was to determine if GlyRs and/or GABA_A_Rs mediate auditory PPI in the decision-making neurons that initiate startle, the Mauthner-cells (M-cells). Our primary findings indicate that GABA_A_Rs function as effector mechanisms mediating the onset and peak effect of PPI, corresponding to interstimulus-intervals (ISIs) ranging from 20–100 ms. GlyRs, in contrast, are primarily involved in the mediation of fast-onset feed-forward (sound-evoked) inhibitory processes that rapidly decay but overlap and contribute to the onset of PPI (20 ms). Independent of these distinct roles in sound-evoked inhibition, GABA_A_Rs and GlyRs both act as mediators of tonic inhibitory processes that modulate M-cell excitability, causing corresponding shifts in the magnitude and duration of sound-evoked excitation.

### GABA_A_Rs Mediate Tonic Excitability and Sensorimotor Gating

We found that inhibition elicited by auditory prepulses was unambiguously abolished or reduced after treatment with bicuculline. Specifically, we found that PPI was reduced at all ISIs, but the effect of the GABA_A_R antagonist was most prominent at ISIs from 20–100 ms, corresponding to the time-course of peak inhibition, i.e., where PPI is strongest. At longer ISIs, the reductions in PPI in bicuculline conditions relative to controls were not statistically significant (*P* > 0.05); however, this may be mainly due to the generally weak effect of PPI at long ISIs. We interpret the failure of PPI after pharmacological blockade as direct, *in vivo* evidence that GABA_A_Rs function as critical effector mechanisms mediating PPI at the level of the M-cells. The effects observed here can conceptually be understood as a form of long lasting GABAergic “feed-foward” inhibition that works in concert with the fast acting and fast decaying glycinergic feed-forward inhibition that has been previously described in the M-cell (reviewed in Korn and Faber, 2005). The identity of the GABAergic inhibitory neurons mediating the longer lasting inhibition associated with PPI in the M-cell is not yet known. In the startle circuit of rodents, however, PPI is at least partly mediated by upstream midbrain and forebrain inhibitory pathways (reviewed by Yeomans et al., 2006). Given the conserved nature of startle circuits in vertebrates, we raise the possibility of comparable extrinsic upstream PPI circuits in fish (Curtin et al., 2013).

### Glycine Receptors Mediate Tonic Excitability and Feed-Forward Inhibition

In contrast to the effects of bicuculline, strychnine had no general effect on PPI, but did cause an ISI-specific reduction in PPI effects at the shortest inter-stimulus interval (ISI) tested, 20 ms. PPI was fully recovered and comparable to control conditions within 50 ms and for all longer ISIs. Further, even at the 20 ms ISI, the GlyR antagonist never fully abolished PPI effects, suggesting that the GlyR-dependent component of PPI acts in concert with another inhibitory process at this short latency from prepulse onset. Indeed, in experiments with the GABA_A_R antagonists we confirmed the onset of prepulse-evoked GABAergic inhibition at this ISI. These findings indicate a sound-evoked glycinergic process that contributes to the onset of PPI but rapidly decays and does not otherwise contribute to PPI effects. Importantly, though strychnine had little effect on PPI, the GlyR antagonist caused prominent changes in M-cell excitability that were superficially similar to the effects of bicuculline. The lack of effect of strychnine on PPI suggests that the disruption of PPI in bicuculline experiments cannot be attributed to increased excitability in the startle circuit (e.g., as in Curtin et al., 2013).

As with the GABA_A_R antagonist, strychnine treatment significantly increased the peak magnitude and duration of sound-evoked excitation independently of PPI; importantly, some of these effects were time-dependent relative to the onset of the sound stimulus. That is, although strychnine increased the peak magnitude of sound-evoked excitation in the earliest phase of auditory processing (EPSP), prior to the onset of feed-forward inhibition, the enhancement of excitation during the mixed-PSP phase that includes inhibition was dramatically greater. The differential enhancement of the EPSP relative to the PSP reflects the disruption of distinct processes; namely, a tonic inhibition that persistently modulates excitability, and a sound-activated phasic inhibition that significantly influences the magnitude and time-course of the sound response, i.e., the temporal fidelity of auditory processing. In additional experiments we showed that the magnitude of antidromically-evoked APs increased after strychnine treatment, indicating a general increase in M-cell excitability consistent with the enhancement of the EPSP. Tonic glycinergic inhibition of the M-cell has been characterized in previous studies (Korn and Faber, 1990; Faber and Korn, 1998; Hatta and Korn, 1999), and Hatta et al. (2001) additionally linked blockade of tonic inhibition by bicuculline to changes in AP-magnitude, as shown in the present study. Our results thus emphasize a potential functional connection between tonic inhibition and auditory processing.

Our results show that feed-forward inhibition and PPI are overlapping phenomena; however, they are functionally distinct and operate over different times-scales. Namely, feed-forward inhibition, like PPI, is recruited by weak auditory stimuli; unlike PPI, feed-forward inhibition attenuates sound-evoked excitation, generally, rather than selectively inhibiting responses to subsequent stimuli. Experiments testing auditory responses without prepulses showed that the onset of sound-evoked glycinergic inhibition occurs during sound-evoked excitation; further, in PPI experiments, we showed that sound-evoked glycinergic inhibition persists to 20 ms but is decayed within 50 ms. Thus, put plainly, sound-evoked glycinergic inhibition is recruited too early and decays too rapidly to mediate PPI, but this time-course is entirely consistent with the well-characterized primary (disynaptic) feed-forward inhibitory pathway (Korn et al., 1982; Lin and Faber, 1988b; Faber et al., 1989; Medan and Preuss, 2011). Indeed, previous reports have also reported that strychnine blocks auditory feed-forward inhibition (Diamond et al., 1973; Lin and Faber, 1988b; Weiss et al., 2009), but the present study is the first to demonstrate that glycinergic feed-forward processes contribute to the summation of inhibition during PPI. In experiments with strychnine, we showed that PPI was reduced but not abolished, indicating an additional inhibitory component; we subsequently identified this as a GABA_A_R-dependent process (see above). Our results thus emphasize the likely contribution of multiple inhibitory pathways in the mediation of PPI. These findings should be interpretated cautiously, however, as the selective effects of the antagonists used are dependent on their effective concentrations, which could not be confirmed in the intact, *in vivo* preparations used in these experiments.

### Mediators, Modulators, and Model Systems

These results highlight some striking similarities with advances in rodent model systems. Yeomans et al. (2010) showed that bicuculline disrupts the peak inhibitory components of behavioral PPI in rodents, and that PnC neurons (the sensorimotor interface equivalents of the M-cell in the mammalian startle circuit) are inhibited by GABA in an *ex vivo* brain-slice preparation. Moreover, the time-course of PPI mediated by GABA_A_Rs reported in rodents is similar to the ISI-specific effects we report here in the fish startle system.

Our findings are somewhat in contrast with studies of glycinergic inhibition in rodent preparations. Koch and Friauf (1995) showed that local and systemic applications of strychnine had no effect on phasic inhibitory processes including short-term habituation of startle and PPI. Geis and Schmid (2011) used *in vitro* patch-clamp recordings to demonstrate that glycine directly inhibits PnC neurons in a rat brain slice preparation; however, they found no evidence that GlyRs were involved in phasic inhibitory processes, including feed-forward inhibition and short-term synaptic depression.

In contrast, our experiments identified GlyR-dependent phasic inhibitory processes that attenuate sound-evoked excitation in multiple contexts (Figures 1, 2). These contrasting findings may reflect underlying differences in goldfish and rodents, or in experimental preparations or stimulus protocols. Whereas the present study measured *in vivo* synaptic processes in mature, awake goldfish, *in vitro* slice preparations used to record from PnC neurons were derived from embryonic rat brains (Yeomans et al., 2010; Geis and Schmid, 2011). Given the profound structural and functional shifts attributed to GlyRs and GABA_A_Rs during development, functional differences between mature and embryonic circuits may be expected (Ehrlich et al., 1999; Nabekura et al., 2004).

In sum, this study characterized *in vivo* synaptic signaling mechanisms that directly mediate the balance of excitation and inhibition at the sensorimotor interface of the startle circuit. Prior studies in the M-cell and other model systems have examined the role of neuromodulators, particularly monoaminergic transmitters (Medan and Preuss, 2011; Curtin et al., 2013), involved in PPI. Our results emphasize that *in vivo* electrophysiological methods can be applied to dissect overlapping inhibitory processes and effector mechanisms to directly test predictions drawn from advances in other model systems. Thus the M-cell presents an appropriate tool for dissecting the functional roles of synaptic processes as well as the effector mechanisms mediating their effects.

## Conflict of Interest Statement

The authors declare that the research was conducted in the absence of any commercial or financial relationships that could be construed as a potential conflict of interest.
